# Inspired by vitamin A for anti‐ageing: Searching for plant‐derived functional retinoid analogues

**DOI:** 10.1002/ski2.36

**Published:** 2021-05-27

**Authors:** N. J. Sadgrove, J. E. Oblong, M. S. J. Simmonds

**Affiliations:** ^1^ Jodrell Science Laboratory Royal Botanic Gardens, Kew Richmond UK; ^2^ Mason Business Center The Procter & Gamble Company Mason Ohio USA

## Abstract

**Background:**

Cosmetic treatments that inspire one's appearance to resemble their younger portrait often utilize ingredients that confer acute effects, particularly hydration by creating hydrophobic barriers or transient elevation of barrier water content. But superior therapies successfully promote morphogenesis of the dermal‐epidermal junction, inspiring extracellular matrix (ECM) formation. This can be achieved by agonism of the very well‐known retinoid nuclear receptors using the endogenous ligand all‐*trans* retinoic acid (tRA), tRA precursors or plant‐based functional analogues, with reduced side effects.

**Aims, Materials and Methods:**

While there are already many promising cosmetic ingredients available from the world's flora, higher potency is favoured, so increasing known candidates is a worth undertaking. Functional analogues of retinoic acid can be identified by culturing fibroblasts with lipophilic candidates from the plant kingdom and assessing gene‐arrays. Modern approaches to validating these findings include the coculturing of fibroblasts with keratinocytes as a measure to predict the potential effects of crosstalk.

**Results and Discussion:**

In this regard, the most promising plant‐derived candidates are of terpene or meroterpene origin, including derivatives of squalene and phytol. Surprisingly pimaric or abietic acids and labdane diterpenes are also noteworthy agonists of the retinoic acid receptor, stimulating collagen expression in dermal fibroblasts.

**Conclusion:**

There are numerous derivatives of these terpenes available from the world's flora and research conducted thus far encourages further screening of these chemical candidates.

## Graphical abstract

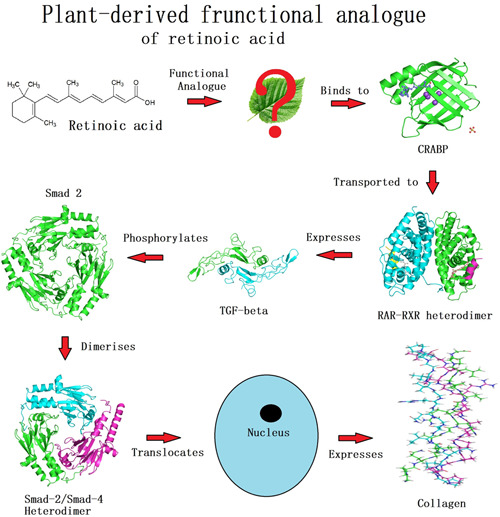

1

**What is already known about this topic?**

- Retinol is a key ingredient in many cosmetics for its' anti‐ageing properties, but it can cause irritation. Therefore, there is an interest in finding new leads.
- Fibroblasts are key targets for reversing or slowing the progress of dermal ageing and have been used in many studies to identify anti‐ageing leads from plants.
- Review highlights the mode of action of some known plant‐derived anti‐ageing compounds such as squalene, bakuchiol and phytol.

## 2 INTRODUCTION

**FIGURE 1:**
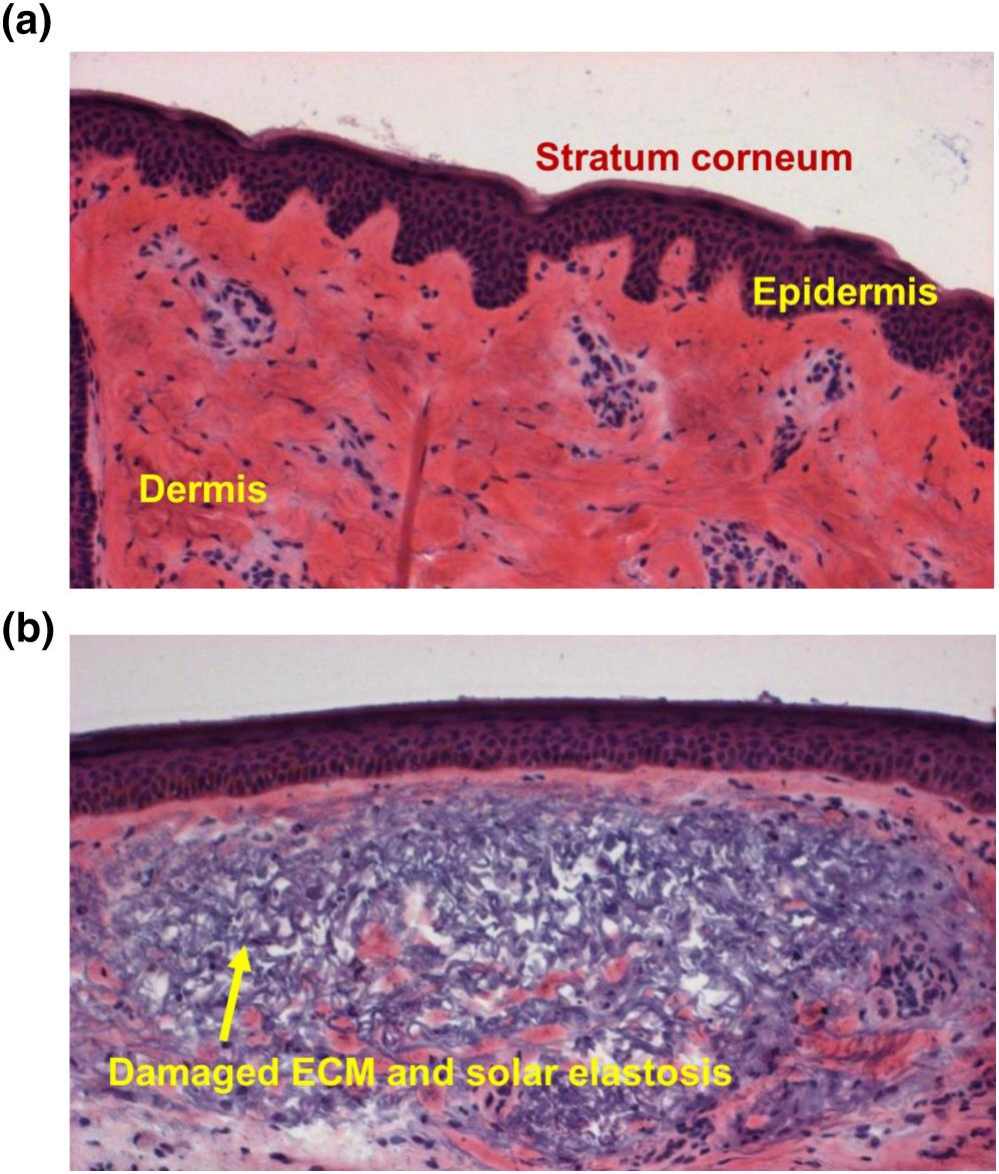
Haematoxylin and eosine stained histologic section of skin from female face. (a) Normal healthy skin from 23‐year old shows features of three major layers of the skin and intact ECM in the dermis (red colouration). (b) Photodamaged skin from a 73‐year old shows significant damage to the ECM and solar elastosis (grey colouration). Image taken from clinical study described in Kimball et al.^8^

As a reflection of a natural and healthy chronological progression, the signs of ageing can be perceived as defining wisdom. However, over the course of one's lifetime, extrinsic factors can damage the epidermis and dermis of skin (Figure 1) and alter its course during the ageing process. Dermal ageing in particular is a process that can occur either intrinsically (chronological) or extrinsically, where lifestyle factors promote the progression of dermal extracellular matrix (ECM) degradation,^1^ leading to the formation of fine or deep lines, mottled discolouration, loss of elasticity and slower recovery from injury.^2^ Protection against these extrinsic factors and ameliorating their long‐term effects is considered a necessary part of physical and psychological health; both are included in the definition of ‘successful ageing’.^3^ The extrinsic factors that negatively influence ‘successful ageing’ include stressful lifestyles, poor eating habits, smoking, chemical exposure and most importantly, UV‐induced damage such as that caused by elevated matrix metalloproteinase expression.^4^, ^5^ For example, despite a paradigm shift in the tanning philosophy, extrinsic UV‐induced damage has already afflicted a cohort of people from the ‘sunbathing paradigm’ who are now entering and passing mid‐life.

Today, people of all ages are turning to the cosmetics industry hoping to persuade their complexions to be more consistent with their intrinsic age, or even younger if possible. The cosmeceutical options vary and research continues to provide new and powerful solutions. Many of the variations of dermal treatments involve unpleasantries, such as invasive surgeries and laser or chemical peels. This encourages researchers to look for more aesthetic alternatives, but this approach is met with varying success. For example, a suitable synthetic alternative to the classic retinoic acid precursor retinol (vitamin A) has not been found. Alternatives are highly sought after, because retinol is well known to induce pronounced irritation in many people by agonism of the ‘transient receptor potential channel vanilloid subtype 1’ (TRPV1), which is an irritant receptor for capsaicin.^6^

In aged skin, collagen fibrils are fragmented and coarsely distributed and the relative amount of collagen‐3 starts to increase, which is merely a consequence of the continued degradation of collagen‐1,^7^, ^8^ the fibril forming collagen.^1^ Due to the progressive degradation of the ECM in aged skin, dermal fibroblast attachment is impaired, leading to reduced size and senescence.^4^ This antagonises the fibroblast's normal function, which is to secrete the matrix proteins, collagen, elastin and proteoglycans, following activation by various cytokines, such as TGF‐*β*1^1^ in the presence of endogenous ligands.

Fibroblasts are also at the interface between the dermis and epidermis, mediating adhesion between the two layers and providing mechanical binding and spreading.^4^, ^5^ The physical interaction is necessary for the fibroblast to maintain essential cell functions. Degradation of collagen and attenuation of the physical interaction of fibroblasts with collagen fibrils, inevitably leads into expression of reactive oxygen species and matrix metalloproteinase‐1, which positively reinforces degradation of the ECM and re‐expression of the previous two effectors and so the cycle continues.^9^ Thus, the fibroblast constitutes the most important target in reversing or slowing the progress of dermal ageing. However, research is also conveying an essential dialogue between the fibroblast and keratinocyte, which involves complex signalling dialogue that challenges the gene expression patterns observed in single fibroblast cell cultures.

Screening plants extracts may identify ligands with functional overlap between plants and mammals. For example, due to the omnipresence of opportunistic microbes in the world, plants and animal pathogen receptors have converged^10^ but this does not explain convergence of growth and cell activation factors. Nevertheless, functional analogues of endogenous agonists and small peptide cytokines are already known, where ligands in the plant kingdom are also ligands in mammals, but specificity and exact roles are different.^11^, ^12^, ^13^ More importantly, ligands that promote growth and cellular activity in plants sometimes function as signals that cascade into collagen expression and secretion in mammalian dermal fibroblasts. While this encourages us to look further into the plant kingdom for biomimetic molecules, a growing interest in natural molecules as replacements for synthetics in the consumer market is what gives impetus to this approach.

Growth and repair of the dermal ECM is mediated in conjunction with fibroblast cell growth and gene‐expression. For example, agonism of the dermal fibroblast retinoid receptors (RAR‐*α*, ‐*β*, or ‐*γ*) and their heterodimers (RAR‐RXR) stimulate the expression of kinases. This triggers a complex orchestration of cellular transcriptions, a cross‐talk between dermal fibroblasts and keratinocytes and dermal ECM morphogenesis.^14^ This makes the classical view of mere collagen synthesis inadequate and conveys the importance of a prime mover, such as retinol, in wholistic dermal rebuilding. Thus, the current review summarizes much of what is known about plant‐based anti‐ageing therapies that target the RAR receptor. What is clear is that plant‐based functional analogues of vitamin A are not necessarily structurally related to this endogenous ligand. Surprisingly, many promising candidates from the plant kingdom have no structural resemblance at all, such as the labdane or pimarane acids. Nevertheless, the research outcomes of many studies in the literature convey implicitly the chemical families that are worthy of further investigation.

### 2.1 Molecular mechanism of extracellular matrix formation

Human skin is comprised of three major layers, the epidermis, the dermis (Figure 1) and the hypodermis. The outermost layer, the epidermis, is a general indicator of health, but this layer also conveys the plight of the deeper layers. Specifically, fine lines, wrinkles, loss of skin elasticity and uneven surface derive from beneath the epidermis, the dermis. Some cosmetics aim to hide these symptoms by promoting skin hydration to increase firmness and create a filling effect. These compositions focus on the glycosaminoglycans either by promoting rejuvenation or by physically injecting them into the dermis (hyaluronic acid fillers). Alternatively, therapies also seek to promote the rebuilding of the ECM by switching on the expression of genes within the fibroblasts to promote secretion of the protein and proteoglycan building blocks.

At the basement membrane, junction between the dermis and epidermis is a high density of fibroblast and keratinocyte cells. As the name suggests, keratinocytes secrete keratin in the epidermis, which provides adhesion between cells and creates a protective layer. The fibroblast secretes the proteins, glycosaminoglycans and proteoglycans that build the ECM of the dermis. The major protein in the dermis is collagen‐1 which constitutes about 85%–90% of the protein fraction of the ECM. The main glycosaminoglycan component of the ECM is hyaluronic acid. Thus, the classical approaches towards dermal rejuvenation targeted the expression of collagen‐1 and hyaluronic acid. However, in fibrotic conditions, such as scars, keloid scars and fibrosis or sclerodermatitis, inhibition of the expression of these proteins is regarded as a therapeutic intervention.

In dermal fibroblasts, the prevailing retinoic acid receptor subtypes are RAR‐*α* and RAR‐*γ* ^15^; each of which can form a heterodimer with the RXRs. Expression of the battery of target genes is initiated by agonism of the RAR‐RXR heterodimer by the endogenous ligand tRA, which only requires binding affinity for the RAR receptor, not RXR. In the cytoplasm, tRA and 11‐*cis* RA bind to the intracellular lipid binding protein CRABPII, which creates a complex that channels the respective RA ligand into the nucleus for complexation with the nuclear receptor RAR, leading to the induction of a retinoid response program of RAR‐target genes that regulate such cellular functions as growth, differentiation and metabolism.^16^ Gene expression profiling has identified numerous genes that are increased by tRA exposure. Gene associated with development, such as lipid and retinoid metabolism, were increased by tRA treatment in human skin explants.^17^ Kong et al.^18^ found that COL1A1 and COL3A1 were both upregulated in human skin after 4 weeks of treatment under occlusion. Future work is needed, taking advantage of the microarray capability but with caution due to the variability that can be driven by tRA.^19^

Activation of RAR may also target other non‐canonical signalling cascades such as protein kinase b (Akt) or extracellular signal regulated kinase (ERK), in modulation of ECM proteins.^20^, ^21^ Additionally, it has also been discovered that RAR*α* resides at the plasma membrane in lipid rafts and upon tRA binding leads to the activation of the p38 mitogen‐activated protein kinase (p38MAPK) signalling pathway.^22^ It is also known that expression of the genes involved in dermal ECM rejuvenation is mediated in part by transforming growth factor beta type 1 (TGF‐*β*1). Expression of this growth factor could also potentially be induced by agonism of the retinoic acid receptors. Since it has been reported that tRA‐mediated gene expression involves triggering a p38MAPK signalling cascade by Tanoury et al.^23^ and that p38MAPK is involved in inducing expression of TGF‐*β*1,^24^ it is possible that part of tRA induction of ECM‐related genes includes further amplification via the TGF‐*β*1 signalling loop.

TGF‐*β*1 is secreted extracellularly, activated by proteases and binds to its endogenous membrane receptor (TGF receptor 2). This initiates recruitment, *trans*‐phosphorylation and activation of ‘activin receptor‐like kinase 5’ (signalling receptor‐1) that can phosphorylate the transcriptional factors, Smad2 and Smad3 (Smad2/3). Smad2 and SMad3 can dimerize with Smad4, yielding Smad2‐Smad4 and Smad3‐Smad4 heterodimers that translocate into the nucleus and stimulate target gene expression.^25^ Target genes include the collagen genes COL1A1 and COL1A2, which encode for the two major components of Type 1 collagen, pro‐*α*1(I) and pro‐*α*2(I).^7^ Other target genes include the proteins, proteoglycans and glycosaminoglycans involved in building the ECM. Furthermore, TGF‐*β*1/Smad signalling downregulates the expression of matrix metalloproteinases (MMP), which are responsible for digestion of the ECM. Thus, the TGF‐*β*1/Smad signalling pathway is very important for building the ECM.^4^

The TGF‐*β*1 and Smad2/3 signalling cascade is not the only process leading to collagen expression. It has been demonstrated that a G‐coupled protein receptor, the cell membrane receptor adenosine A_2A_, also conditionally modulates expression of collagen synthesis.^26^ The cascade is modulated in a concentration dependent transactivation process, where full agonism leads to the upregulation of its principle effector, cyclic adenosine 3′,5′‐monophosphate (cAMP), which promotes TGF‐*β*1 and Smad2/3 signalling and hence collagen‐3 synthesis, inhibiting collagen‐1 synthesis and leading into fibrosis or scar tissue formation. Thus, only partial agonism of the receptor, or a lower concentration of the agonist, is regarded as cosmetically viable. Partial agonists of the adenosine A_2A_ receptor modulate a lower expression of cAMP, which enables full expression of both of the collagen types (Col‐1 and Col‐3) and also sees fibroblast proliferation. This allegedly occurs independent of Smad2/3 and is alternatively mediated by Akt, as mentioned earlier. This means that the adenosine A_2A_ receptor may be a concentration dependent switch for TGF‐*β*1‐induced Akt mediated collagen‐1 expression.^26^

TGF‐*β*1 also regulates the expression of various MMP. The MMPs are a zinc‐dependent family of metalloendopeptidases responsible for degradation of the ECM.^27^ In fibroblasts, TGF‐*β*1 inhibits the expression of MMP‐1 and promotes expression of MMP‐2.^28^ MMP‐2, also known as gelatinase A, has the ability to degrade gelatin, all the collagen types, elastin, fibronectin and laminin, just to name a few^29^; however, the collagenase activity of MMP‐2 is much weaker than MMP‐1.^30^ Thus, promotion of MMP‐2 and suppression of MMP‐1 positively influences ECM rejuvenation.

The proteolytic turnover of components in the ECM is a natural process that is ongoing, but is upregulated subsequently to UV or mechanical insults, leading to tissue remodelling. For example, digestion of a matricellular protein by MMP‐3 produces polypeptides that promote angiogenesis.^31^ In the same way, it is possible that the activity of MMPs on collagen yields collagen degradation products that signal for the transcription of replacement collagen. It has been demonstrated that oral digestion and specific metabolism of collagen peptides can be associated with increases in the glycosaminoglycans and collagen in the dermis. Furthermore, small peptide metabolites were identified as the active principle leading to collagen synthesis by triggering the TGF‐*β*1/Smad signalling cascade and by reducing the activity of MMP‐1, MMP‐3 and AP‐1 ^32^. In aged skin, AP‐1 induced by reactive oxygen species inhibits the TGF‐*β*1/Smad signalling cascade in dermal fibroblasts.^4^ Finally, it has been reported that pre‐treatment with topical tRA can prevent UV‐induced MMP‐1, ‐3 and ‐9 expression in human skin.^33^

Another potential therapeutic target in anti‐ageing therapies that stimulate the expression of collagen are the peroxisome proliferator‐activated receptors (PPARs). It has been demonstrated that agonism of PPAR‐*γ* downregulates collagen expression.^34^ Conversely, antagonism of the same receptor promotes collagen expression.^35^ The other receptor types have opposite effects. Agonism of PPAR‐*β*/*δ* was reported to increase collagen types I and III and fibronectin expression in human dermal fibroblasts, which demonstrates the importance of selectivity.^36^ It has been shown that agonism of PPAR‐*β*/*δ* is another part of the function of retinol,^23^, ^37^ which has no measurable affinity for PPAR‐*γ*. Further work is merited in better understanding the differentiating effects between RAR and PPAR‐*β*/*δ* regulation of ECM related genes.

The pleiotropic effects of tRA and its high selectivity means that identifying functional analogues is no small task. A functional analogue may or may not have high selectivity for the RAR or RXR receptor and PPAR‐*β*/*δ*. But true functional analogy occurs with similar gene expression profiles.^38^ Furthermore, partial agonists of the adenosine A_2A_ receptor appears to conform to structural analogues of adenosine but with lower degree of agonism. In the context of peptides, specific metabolites of digested collagen were identified as important mediators of replacement collagen synthesis, but the presence of other peptides did not interfere with this activity.

Numerous studies have identified plant‐derived functional analogues of the above‐mentioned endogenous ligands. Research leads have been garnished from a historical synopsis of botanical extracts used in dermatological treatments, determined empirically over the course of iterative trial and error selection, potentially spanning hundreds or thousands of years, such as pomegranate.^39^ However, much insight has been gained on structural pre‐requisites and this has hastened the process of identifying suitable cosmeceutical candidates by revealing metabolites not previously recorded in human use for such applications.

### 2.2 Plant ‘skin’: their lipids and lipophilic terpenes as RAR agonists

Because the binding domain of the RAR receptor is lipophilic and is a nuclear receptor, where only passage is granted to lipophilic membrane penetrating molecules, it makes sense that plant lipids, aliphatic linear terpenes and their derivatives have potential as functional analogues of retinoic acid. According to modern scientific opinion, it is not a coincidence that such compounds are present in the epidermis of plants at high concentrations. As a protective organ, the most prominent barrier function of the ‘plant skin’ is to delay moisture loss and filter the sun's rays to attenuate negative UV‐induced damage. However, it is a coincidence that many of the plant's terpenes and lipids can modulate the expression of genes coding for human proteins, including dermal collagen. For example, a fatty acid derivative known as ‘traumatic acid’ was isolated from *Phaseolus vulgaris* in 1939 by American chemists and identified as a potent plant—wound‐healing compound.^40^ The etymological genesis of traumatic acid is evidently related to the expression in injured plant‐tissue. Recently, it has been shown that traumatic acid promotes collagen expression in cultured human fibroblasts and dramatically attenuates reactive oxygen species generation,^41^ oddly conveying a translation of wound healing potential to mammals. In light of this, many more lipid derivatives are known and more are being discovered in the plant kingdom, mainly as hydroxylated polyunsaturated derivatives of common essential free fatty acids such as linolenic or linoleic acid,^42^ as well as omega‐3 and palmitoleic. Inspired by the previous example, such fatty acid derivatives should be examined in the context of RAR agonism and anti‐ageing effects to the dermis.

In the current cosmetics field of practice, fixed plant oils are frequently incorporated into skin care products and regimens, but not merely for their aesthetic effects. Skin ageing and chronic UV exposure alter the expression of fatty acids in the epidermis,^43^ making aged skin feel dry. Oils like safflower or argan oil and a host of others^44^ serve as barriers against dehydration and may be involved in downregulating MMP‐1 following UV‐induced damage. Furthermore, modern cosmetics seek to utilize therapeutic ingredients in oils, such as phospholipids or squalene, to enact anti‐inflammatory and antioxidant effects. Squalene is an unorthodox triterpene because it is aliphatic and acyclic; constructed out of two ‘tail‐to‐tail’ sesquiterpenes (not head‐to‐tail). It is found in olive oil or many different plant species and is also produced by marine microalgae. Moreover, it is an agonist of PPAR‐*α*,^45^ indicating a role in lipid metabolism, ceramide synthesis and keratinocyte proliferation. In the cosmetics industry it is hydrogenated to squalane to improve shelf life without loss of positive effects.

As compared to squalene, similar types of triterpenes can be found in the plant kingdom that have not been examined in the context of RAR agonism and promotion of gene expression profiles to emulate tRA. For example, the plant kingdom furnishes human kind with many hydroxy derivatives of squalene, such as the cosatetraenes from the South African species *Ekebergia capensis*.^46^ Furthermore, a smaller terpene chain, phytol, which is a diterpene, has been implicated as part of a lipophilic mixture in effects consistent with RAR agonism and collagen building. The complete mixture is extracted from an important South American herb known in English as ‘Black Jack’ (*Bidens pilosa* L.), which is composed of the lipids, linolenic, linoleic, oleic and palmitic acid and of course the terpene phytol.^47^ Key to this extract is the balance of ingredients, particularly oleic acid. No two oils are the same—olive oil is thought to provoke contraindication if applied too frequently due to the greater abundance of oleic acid,^44^ but the oil of ‘Black Jack’ is evidently not predisposed to this problem.

Lipids are more frequently considered in the context of epidermal cells and keratinocytes, but they are also of relevance in the modulation of PPAR receptors, promoting ceramide expression and incorporation into the lipid bilayer of the epidermis, enhancing the hydrophobic barrier. The subsequent increase in ceramide levels as promoted by specific free fatty acids may augment fibroblast gene expression. There is currently no concrete evidence to support this specifically in dermal cells, but other cells display effects that motivate for a closer examination, particularly by focusing on exogenous plant‐based derivatives. While ceramides are generally regarded as ingredients that restore the skin barrier and participate in cellular signalling,^48^ ceramide itself has been demonstrated to confer modulatory effects to the alpha isoforms of the RAR‐RXR heterodimer gene battery in osteoblast cells.^49^ Ceramides are also well‐known agonists of the PPAR membrane receptors, but it is highly likely that research efforts focused on ceramide derivatives from nature may reveal molecules with unique binding abilities to fibroblast transcription factors. While the same could be said for sphingoids or sphingosine bases, sphingosine derivatives are also implicated in fibrogenesis pathologies in myofibroblast cells of the liver.^50^ While this conveys the potential to promote collagen expression in some contexts, it is necessary to prove safe usage in topical applications. Nevertheless, this observation highlights the need to assay sphingoid base derivatives. In the African continent many ceramides and unique fatty acids can be isolated from the rich botanical aggregate, particularly from species endemic to the tropical sub‐Saharan forests of central and west African countries,^51^ including ceramides like triumfettamide and triumfettoside Ic from *Triumpheta cordifolia* A. Rich.^52^ Hence, there are many prospective candidates awaiting testing.

### 2.3 Agonists of RAR from the plant kingdom

Dietary retinol is obtained in the form of retinyl palmitate from animal sources and as *β*‐carotene (provitamin A) from vegetable sources. *β*‐Carotene is metabolized to retinol and retinal and distributed through the body via chylomicrons. Retinyl palmitate is the predominant storage form of retinol that provides a source when needed. If there is a cellular need for tRA, this ester is hydrolysed to *trans*‐retinol which in turn is oxidized to *trans*‐retinal and, subsequently, to the active form tRA. Levels of tRA are tightly regulated through the action of cytochrome P450s that metabolize tRA to 4‐oxoRA, a retinoid inactive metabolite.^53^ Several isomers of tRA are derived in metabolism and have *cis* configurations either at bonds 9, 11 or 13 (9‐*cis* RA; 11‐*cis* RA or 13‐*cis* RA).^54^

Interestingly, tRA is also an agonist of the PPAR‐*β*/*δ* nuclear receptor.^55^ It has been reported that in this case the intracellular fatty acid binding protein 5 transports tRA into the nucleus, leading to the PPAR‐*β*/*δ* regulated expression of other subsets of genes responsible for homeostasis and the insulin response.^23^ The activation of either RAR or PPAR‐*β*/*δ* by tRA is dependent on the differential binding and portioning between either CRABPII or fatty acid binding protein 5, respectively, that dictates which gene expression program will be initiated.^56^ Transport proteins also bind other lipophilic compounds and transport them to cognate receptors, which includes plant‐derived metabolites. There are many known botanically sourced compounds that elicit similar gene expression profiles as tRA. The classic example is allantoin, which has demonstrated fibroblast proliferation and collagen synthesis in vitro.^57^ However, sometimes similar gene expression profiles as compared to retinoic acid can be elicited without acting on the same nuclear receptor, as is illustrated in the following example.

A plant‐based functional analogue of retinol is evident from the research backing the use of *Psoralea corylifolia* L. [synonym of *Cullen corylifolium* (L.) Medik: Leguminosae] as an anti‐ageing natural cosmetic. Despite having some fundamental structural differences, bakuchiol evoked a similar gene expression profile to retinol,^58^ including upregulation of the lipid binding CRBP transport proteins, suggesting the possibility of specialized cellular uptake. Other genes that were upregulated above the level of retinol included ones associated with extracellular matrix and dermal‐epidermal junction content. Gene upregulation was achieved without promoting cell proliferation.^58^ While the gene expression changes suggest bakuchiol can mimic a retinol‐like response, further mechanistic work is required to establish whether it functions via direct RAR activation or alternate pathways such as retinoid metabolism.

Lipophilic agonists of all the retinoid receptors should be investigated for their potential to upregulate the expression of the battery of genes associated with extracellular matrix rejuvenation and repair, including downstream effects. However, finding an agonist for a transcriptional factor does not mean that transcription is going to resemble that of endogenous agonists. Binding affinity of a drug and intrinsic efficacy are treated as two independent outcomes of receptor agonism.^59^ Transactivation of genes is proportional to the intrinsic efficacy of a ligand. Furthermore, there is increasing evidence that partial agonists may lead to different expression profiles of the battery of genes with common *cis*‐regulatory elements. This has been demonstrated through examination of the partial agonists of PPAR‐*γ*.^60^

Thus, agonists of receptors should be examined in conjunction with their gene expression profiles.^38^ By following this approach, unsurprisingly it has been demonstrated that several naturally occurring RAR agonists demonstrate different gene expression profiles. Specifically, lipophilic pimarane diterpenes from the rhizome of *Aralia cordata* (pimaric, pimaradienoic acid and abietic acid) were found to be novel agonists of RAR but elicited distinct differences in gene activation as compared to retinol.^61^ Furthermore, another natural product luffariellolide was also demonstrated to be an RAR agonist, but with a unique binding mode and an unexpected covalent modification on the RAR, which again elicited a distinctly different gene expression profile.^62^ Alternatively, a search for natural RAR agonists that give similar expression profiles to RA is a realistic objective. Synthetic versions have been created that achieve dramatic positive effects on the photoaged ECM in mouse models.^63^

It has been proposed that only slight differences in gene expression profiles may help to overcome issues of RA resistance and create positive outcomes associated with RAR agonism by excluding unwanted side effects. Although this approach in the context of natural products is still only gaining traction in the cosmeceutical sciences, some promising outcomes have already been delivered, such as in the case of bakuchiol. Furthermore, a much more hydrophilic compound ‘lithospermate B’ was able to upregulate the expression of TGF‐*β*1 and hence collagen‐1 and ‐3, possibly by agonism of the nuclear PPAR*β*/*δ* receptor.^64^ This is surprising, because unlike bakuchiol, lithospermate B has a moderate to high polar headspace and nuclear receptors have specialized transport for the more lipophilic endogenous ligands.

Another species that produced metabolites conferring similar gene expression patterns as compared to retinol is *Andrographis paniculata*, but allegedly by targeting epidermal stem cells that promoted vascular endothelial growth factor, cascading into type‐1 collagen synthesis in dermal fibroblasts.^65^ The major diterpene andrographolide, was hypothesized to be active on the RXR receptor.^66^

Andrographolide is a labdane diterpene, lipophilic enough to bind to the lipid binding domain of a nuclear receptor. It is interesting that the labdane has no resemblance to an endogenous ligand. Nevertheless, there is increasing evidence that lipophilic labdane derivatives mediate positive gene expression profiles in the dermal‐epidermal junction by promoting antioxidant defences and expression of cytokines associated with rejuvenation.^67^ Another prominent example is sclareol from *Salvia sclarea* L., which is a labdane‐type diterpene able to upregulate the expression of collagen‐1 and decrease MMP‐1 in cultured fibroblasts, attenuating the effects of UVB‐photo ageing.^68^ Although many other flavonoid and anthraquinone derivatives were tested, the labdane was the only one to demonstrate these effects.

Another abietic acid, carnosic acid from rosemary, demonstrated potent inhibitory effects against UVB induced photo damage, in particular by inhibition of reactive oxygen species generation and MMP‐1, ‐3 and ‐9 expression.^69^ While Park et al.^69^ did not assess the RAR agonism potential of carnosic acid, the attenuating effect against collagen loss and photo‐ageing is evident. This conveys consistency of the observations made by Tanabe et al.^61^ who conveyed that the bicyclic classes of diterpene, abietic, pimaric and pimaradienoic acids are generally RAR agonists. Considering this and the observation made by us of a role for labdanes, it is evident that these bicyclic diterpenes have great potential in anti‐ageing cosmeceuticals. In this regard, there are numerous chemical candidates in the world's flora that can be screened, including the abietane, pimarane and labdane‐rich genus *Croton* (Euphorbiaceae) from Africa,^70^, ^71^ the cosmetic buchu species used by the Khoi‐San people of Southern Africa,^72^ bicyclic diterpenes from Cupressaceae (*Cupressus*, *Callitris* and *Widdringtonia*,^73^ *Chamaecyparis*, *Juniperus, Tetraclinis, Thuja*) and *Olearia* (Asteraceae), including the new Australian species *Olearia fulgens*.^74^ These examples are summarized in Table 1.

**TABLE 1:** Plant species identified as having potential skin anti‐ageing activity

Plant species	Chemistries	Reported mechanism	Reference
*Phaseolus vulgaris* L.	Traumatic acid	Wound healing	40
*Ekebergia capensis* Sparrm.	Cosatetraenes	Anti‐inflammation	46
*Bidens pilosa* L. (‘Black Jack’)	Phytol, linolenic, linoleic, oleic and palmitic acid	Collagen synthesis	
*Triumpheta cordifolia* a.	Triumfettamide, triumfettoside Ic	PPAR agonist	51
*Psoralea corylifolia* L. (synonym of *Cullen corylifolium* (L.) Medik)	Bakuchiol	Retinol mimic	58
*Aralia cordata* Thunb.	Pimaradienoic acid, abietic acid	RAR agonist	61
*Salvia miltiorrhiza* Bunge.	Lithospermate B	PPAR agonist	61
*Andrographis paniculate* (Burm.f.) nees.	Andrographolide	Retinol mimic	65, 66
*Aframomum angustifolium* (Sonn.) K.Schum.	Labdanes	Tissue remodelling	67
*Salvia sclarea* L.	Sclareol	Collagen synthesis	68
*Salvia rosmarinus* Spenn.	Carnosic acid	Collagen synthesis	69
*Croton* L. spp. (Euphorbiaceae)	Abietane, pimarane	Collagen synthesis	70, 71
*Buchu* spp. Cupressaceae (*Cupressus*, *Callitris* Vent.*, Widdringtonia* Endl.*)*, *Chamaecyparis* Spach., *Juniperus* L.*, Tetraclinis* Mast.*, Thuja* L.), *Olearia* Moench (Asteraceae), *Olearia fulgens* A.R.Bean.	Bicyclic diterpenes	Retinol mimic	72, 73, 74

It has been reported that gene expression of keratinocytes and to a lesser degree of fibroblasts, can be modulated by such labdane‐rich extracts (or similar diterpenes). This occurred in the presence of a labdane‐rich extract from seeds of *Aframomum angustifolium*.^67^ Tissue remodelling effects appear to be orchestrated by both cell types, but it is unclear if there is a crosstalk between keratinocytes and fibroblasts or if the labdanes act on both individually.

### 2.4 Crosstalk between keratinocytes and fibroblasts

The crosstalk between keratinocytes and fibroblasts makes the keratinocyte an important participant (or antagonist?). It has been demonstrated that interleukin‐1*α* that is secreted during keratinocyte proliferation blocks the expression of connective tissue growth factor in fibroblasts, while mediating other fibroblast genes,^75^ but these effects are not observed in injured tissues where rapid growth and recovery of the dermis are important.^76^ This means that some therapies that promote keratinocyte proliferation may modulate the process of ECM rebuilding. This is evidently an important consideration for the cosmetics industry.

The use of gene arrays as a means to convey the effects of agonists in fibroblast cell cultures ignores the concurrent effects of the same treatment on keratinocytes. An experimental protocol that may convey a closer approximation of fibroblast gene expression involves the coculturing of fibroblasts with keratinocytes and assessing gene expression in that instance.^75^ It is possible that outcomes in cocultures may more closely approximate in vivo skin environment. Furthermore, mechanisms of cells in monolayer growth may differ from a more wholistic tissue model. Interleukin‐1*α* has both an inhibitory effect on dermal fibroblast collagen synthesis and a stimulatory effect on secretion of hyaluronate.^76^ Since cultured dermal fibroblasts may not respond the same as when in injured tissue, usage of a full thickness skin model, T‐Skin^TM^, may provide a more wholistic approach to assessing the effects of potential ligands on gene expression profiles.^77^

It is worth noting that tRA inhibits the differentiation of epidermal keratinocytes in vitro but can increase in vivo.^45^ In contrast, it induces only a moderate level of proliferation after 24 h by concurrently inducing and suppressing associated genes, with suppression of interleukin‐1*α* after an initial brief period of promotion.^78^ While it appears that functional analogues to retinol involves a balanced response between differentiation and proliferation, it is critical to understand any response profile in both in vitro and in vivo conditions.

It is already known that treatments that upregulate the TGF*β*/Smad pathway in fibroblasts leads into proliferation and ECM reconstruction, but the effects of TGF*β* on keratinocytes are increased expression of MMP and inhibition of cell growth.^28^ This conveys that in the course of natural growth and repair, fibroblast and keratinocyte proliferation or protein expression can be differentially modulated. While fibroblasts are secreting ECM proteins, the keratinocytes are relatively inactive and when the keratinocyte is proliferating, the secretion of interleukin‐1*α* suppresses the protein secretions of fibroblasts and instead promotes secretion of the proteoglycans. This demonstrates the importance of a fluent crosstalk between the two cell layers. Thus, ideally a plant‐derived functional analogue of retinol would supress keratinocyte proliferation temporarily without leading to apoptosis, while promoting ECM protein synthesis at the fibroblast. By strengthening the cellular mechanical tension at the dermal‐epidermal junction, many of the activities of keratinocytes are supported as a consequence, which may be viewed as a ‘bottom‐up’ approach.

## 3 CONCLUDING REMARKS

Plant‐derived compounds are capable of modulating gene expression from the RAR transcriptional factor in a similar way to tRA. This is demonstrated using gene arrays and reporter assays. However, the search for functional analogues of tRA is motivated by the desire to identify a chemical candidate that is capable of promoting collagen expression, extracellular matrix formation and hence anti‐ageing effects without the undesirable irritation effects elicited by tRA or retinol.

Something that has come up in the search for chemical candidates is the mutual exclusion of processes occurring within dermal fibroblasts versus epidermal keratinocytes. This crosstalk is potentially a limiting factor in dermal treatments. It may also highlight the importance of minimizing the complexity of ingredients to avoid countering positive effects derived from single active ingredients. There are many places to start looking for natural product candidates. As previously mentioned, examining gene arrays from whole skin models, such as T‐Skin^TM^ or fibroblast/keratinocyte cocultures, could flag positive candidates. Such candidates may be in the form of retinoid analogues or meroterpenes like bakuchiol. Alternatively, many interesting candidates that have surfaced in the screening process are bicyclic diterpenes, such as pimaric and abietic acids or labdanes. Research efforts to identify more diterpenes have been minimal and a targeted approach is highly optimistic.

## CONFLICT OF INTERESTS

Monique S. J. Simmonds has a grant from Procter and Gamble to support work on the identification of new plant‐derived ingredients. However, this article was not directly funded by them.
